# SLC6A19 inhibition facilitates urinary neutral amino acid excretion and lowers plasma phenylalanine

**DOI:** 10.1172/jci.insight.182876

**Published:** 2024-11-08

**Authors:** Heike J. Wobst, Andreu Viader, Giovanni Muncipinto, Ryan Hollibaugh, Daniel van Kalken, Christopher T. Burkhart, Susan M. Cantin, Rachel M. Bates, Yannik Regimbald-Dumas, Liam Gross, Mitchell T. Antalek, Joshua E. Zweig, Frank Wu, T. Justin Rettenmaier, Matthew T. Labenski, Nicholas Pullen, Heather S. Blanchette, Jaclyn L. Henderson, Haoling H. Weng, Toby A. Vaughn, Dean G. Brown, John P. Throup, Joel C. Barrish

**Affiliations:** 1 Jnana Therapeutics, Boston, Massachusetts, USA.; 2 HW MedAdvice LLC, San Diego, California, USA.

**Keywords:** Clinical trials, Metabolism, Amino acid metabolism, Transport

## Abstract

**BACKGROUND:**

The toxic accumulation of phenylalanine (Phe) in the brain underlies the neurological presentation of phenylketonuria (PKU). Solute carrier family 6 member 19 (SLC6A19) is the major transporter responsible for the (re)absorption of Phe in the kidney and intestine. Here, we describe the characterization of the first small molecule SLC6A19 inhibitor to enter clinical development for the treatment of PKU.

**METHODS:**

C57Bl/6J WT and *Pah^enu2^* mice were dosed with an inhibitor of SLC6A19 to investigate the effects on urinary amino acids and plasma Phe. In a phase 1 study, healthy human volunteers were dosed with JNT-517, an investigational oral inhibitor of SLC6A19. The primary objective of the study was safety. Secondary objectives included pharmacokinetic and pharmacodynamic studies.

**RESULTS:**

Inhibition of SLC6A19 increased the urinary excretion of Phe in a mouse model of PKU, thereby reducing plasma Phe levels. JNT-517, an investigational oral SLC6A19 inhibitor, was found to be safe and well tolerated and increased the urinary excretion of Phe in a phase 1 healthy volunteer study.

**CONCLUSIONS:**

These data indicate that pharmacological inhibition of SLC6A19 presents a promising approach to lower toxic elevated levels of amino acids found in PKU and related amino acid metabolism disorders by facilitating their renal elimination.

**TRIAL REGISTRATION:**

Australian New Zealand Clinical Trials Registry (ANZCTR), ACTRN12622001222730.

**FUNDING:**

The studies in this paper were funded by Jnana Therapeutics.

## Abstract 2

Inhibition of the transporter SLC6A19 may lower toxic levels of the amino acid Phenylalanine in PKU by increasing its urinary excretion.

## Introduction

Phenylketonuria (PKU) is an autosomal recessive disease characterized by the toxic accumulation of phenylalanine (Phe) in the blood and brain. It is the most common genetic disease of amino acid metabolism, with an estimated prevalence of 1:10,000 to 1:15,000 in newborns (1). PKU is caused by deficiency in Phe hydroxylase (PAH), the rate-limiting enzyme in the metabolism of Phe. Left untreated, toxic Phe concentrations cause intellectual disability, cognitive and motor deficits, seizures, and psychiatric conditions including depression, anxiety, and attention deficit disorder (1–3). First-line treatment of patients with PKU is a life-long very restrictive low Phe diet, which is initiated after diagnosis through newborn screening to prevent intellectual disability and neurological and psychiatric impairment (3, 4). There are currently 2 interventional therapies approved for PKU: sapropterin dihydrochloride, the BH_4_ cofactor of PAH that acts as a molecular chaperone in patients with residual enzyme function (3), and pegvaliase, an injectable bacterial enzyme substitution therapy associated with substantial adverse events (5). For the vast majority of patients with PKU, the restrictive nature of the diet is challenging to follow life-long. Despite diet and available treatments, approximately 70% of adult PKU patients have Phe levels exceeding recommended targets of ≤ 360 μM (6–8), and there is a high unmet need for therapies that are safe and effective regardless of *PAH* variant and that allow for diet normalization (7, 8).

Solute carrier family 6 member 19 (SLC6A19, also known as B^0^AT1) is the major transporter responsible for the absorption of free neutral amino acids in the intestine and their reabsorption in the kidney (9). In humans, loss-of-function mutations in *SLC6A19* cause Hartnup disorder (10), a generally benign condition characterized by increased urinary excretion of Phe as well as other neutral amino acids, also referred to as Hartnup amino acids (11). Despite increased renal elimination of neutral amino acids, the majority of patients with Hartnup disorder maintain broadly normal levels of plasma amino acids and are frequently asymptomatic (12, 13). These findings are phenocopied in mice with genetic knockout of *Slc6a19*, which present with aminoaciduria but maintain normal levels of amino acids in the plasma (14). Interestingly, in the *Pah^enu2^* mouse model of PKU, which is characterized by high levels of plasma Phe, homozygous loss of *Slc6a19* causes increased urinary excretion of amino acids including Phe as well as an approximately 70% decrease in plasma Phe (14). Similar to the observations in WT mice, plasma levels of other amino acids are largely unaffected in *Pah^enu2^* mice with loss of *Slc6a19*. This suggests that inhibition of SLC6A19 may be a viable therapeutic strategy to selectively lower toxic Phe levels in patients with PKU.

Here, we describe the characterization of small molecule inhibitors of SLC6A19, identified using a chemoproteomic ligand discovery approach (Reactive Affinity Probe Interaction Discovery, RAPID) (15). Treatment of *Pah^enu2^* mice with a SLC6A19 inhibitor caused widespread aminoaciduria, including increases in urinary Phe, as well as a dose-dependent reduction in plasma Phe. SLC6A19 inhibition was efficacious in *Pah^enu2^* mice across a broad range of elevated basal plasma Phe levels, from classic PKU (Phe > 1,200 μM) to hyperphenylalaninemia (Phe > 360 but < 600 μM). In a phase 1 study in healthy human volunteers, JNT-517, a highly selective, first-in-class, orally bioavailable inhibitor of SLC6A19, liberated urinary neutral amino acids in a dose-dependent manner in single and multiple ascending dose studies. Plasma amino acid levels remained largely within the normal range after 14 days of treatment. These data provide the first clinical proof-of-mechanism that pharmacological inhibition of SLC6A19 mimics the Hartnup phenotype, observed with genetic loss of *SLC6A19* function in humans, confirming the potential for this mechanism to combat the buildup of pathogenic amino acid levels in the context of PKU.

## Results

### Identification and characterization of SLC6A19 inhibitors.

**Figure 1:**
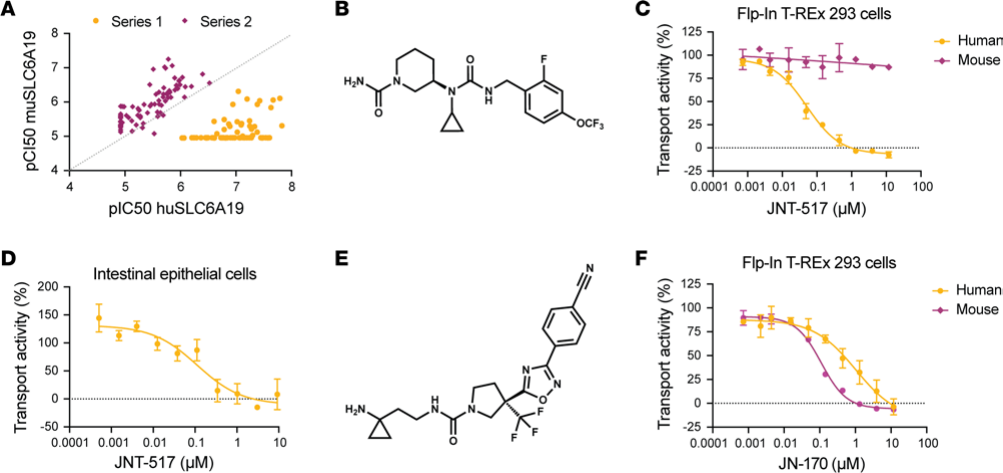
JNT-517 and JN-170 are potent inhibitors of human and mouse SLC6A19. (**A**) Potency of 2 distinct chemical series, series 1 (yellow) and series 2 (purple), against overexpressed human and mouse SLC6A19 in an isoleucine transport assay, expressed as pIC_50_. (**B**) Structure of JNT-517. (**C**) Representative concentration-response curves of JNT-517 in an isoleucine transport assay in Flp-In T-Rex 293 cells cooverexpressing human (yellow) SLC6A19 and TMEM27 or their mouse (purple) orthologs. (**D**) Concentration-response curve of JNT-517 in a glutamine transport assay in intestinal epithelial cells expressing endogenous SLC6A19 isolated from human postmortem intestine. (**E**) Structure of JN-170. (**F**) Representative concentration-response curves of JNT-170 in an isoleucine transport assay in Flp-In T-Rex 293 cells cooverexpressing human (yellow) SLC6A19 and TMEM27 or their mouse (purple) orthologs. Error bars denote SD from 2 (**C** and **F**) or 4 (**D**) technical replicates in a single run. IC_50_ values are geometric means of *n* = 4 (**C**) or 6 (**F**) experiments. For postmortem epithelial transport, IC_50_ is the geometric mean of *n* = 5 experiments from 3 individual donors.

We conducted a high throughput screen (RAPID) assessing the ability of a library of small molecules to displace a reactive affinity probe in a competition binding assay in cells overexpressing human SLC6A19 and its accessory protein TMEM27 (16) (Supplemental Figure 1; supplemental material available online with this article; https://doi.org/10.1172/jci.insight.182876DS1). To confirm functional activity of binders identified, screening hits and structurally related analogs were tested in a SLC6A19 transport assay measuring the inhibitory effects of hit molecules on the uptake of heavy-labeled L-isoleucine, a substrate of SLC6A19. Two chemotypes with potential for drug-like properties, series 1 and series 2, were selected for chemical optimization (Figure 1A). Molecules derived from series 1 were potent inhibitors of human SLC6A19 transport but demonstrated much weaker potency against murine SLC6A19 while analogs from series 2 consistently possessed greater potency against mouse SLC6A19 than the human ortholog (Figure 1A).

Chemical optimization of series 1 ultimately resulted in a clinical candidate, JNT-517 (Figure 1B). The potency of JNT-517 against human SLC6A19 in the isoleucine transport assay was 47 nM (95% CI 38–57 nM) (Figure 1C). However, its functional potency against mouse SLC6A19 was negligible (IC_50_ > 11.8 μM), precluding the assessment of its in vivo efficacy in a mouse model (Figure 1C). JNT-517 also potently inhibited the transport of glutamine in human intestinal epithelial cells with endogenous expression levels of SLC6A19 (IC_50_ = 81 nM, 95% CI 33–197 nM) (Figure 1D). The selectivity of JNT-517 was assessed by measuring its ability to inhibit the amino acid transporters SLC1A5 (17) and SLC7A5 (18) as well as the SLC6 family member SLC6A8, a creatine transporter (19). JNT-517 did not inhibit either SLC1A5, SLC7A5, or SLC6A8 at concentrations up to 35 μM (Supplemental Figure 2). Taken together, these data confirm that JNT-517 is a potent, selective inhibitor of human SLC6A19.

**Figure 2:**
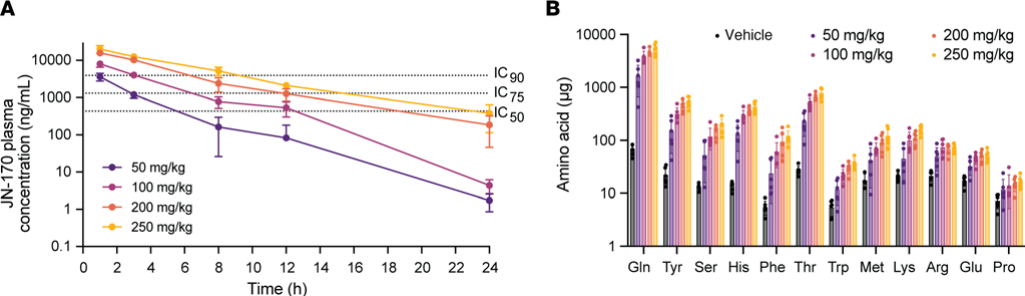
JN-170 causes neutral aminoaciduria in C57Bl/6 mice in a dose-dependent manner. (**A**) JN-170 pharmacokinetics, (**B**) 12 hour urine amino acids after a single oral dose in male C57Bl/6 mice (*n* = 6). All data represent mean ± SD.

**Figure 3:**
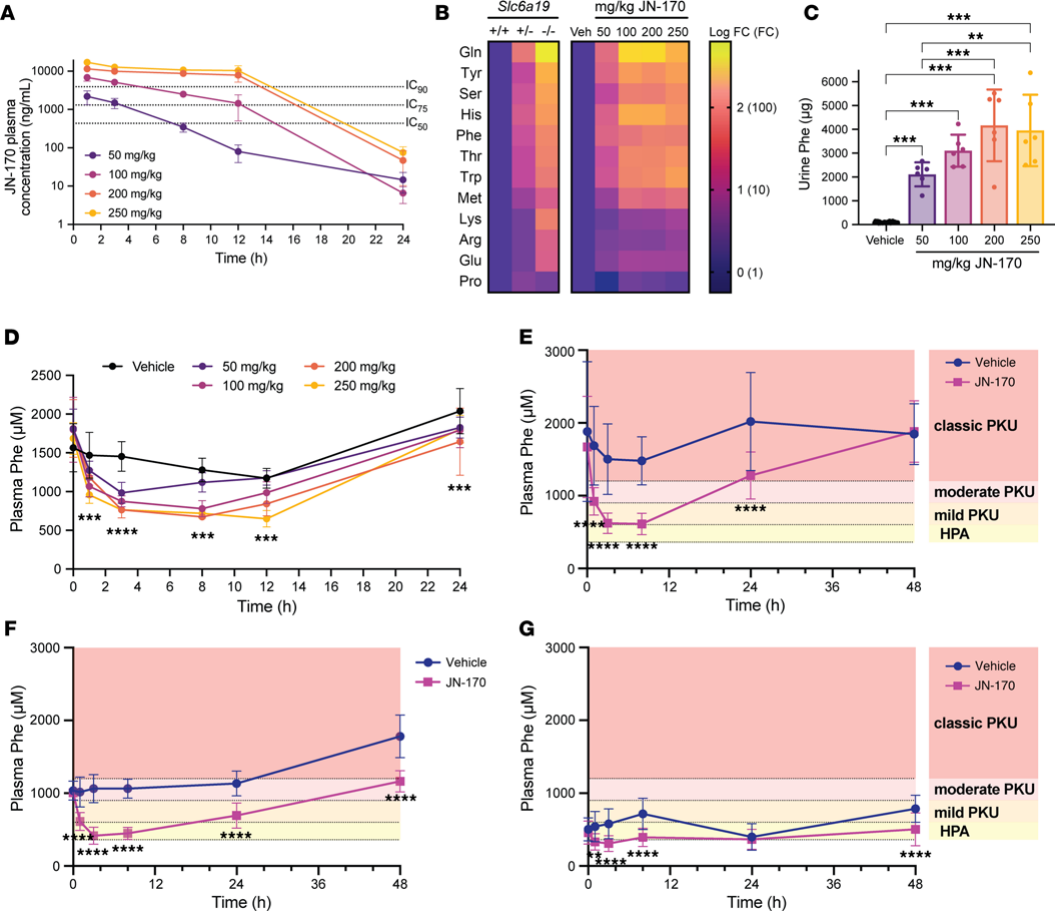
JN-170 lowers plasma Phe in *Pah^enu2^* mice mimicking classic PKU or hyperphenylalaninemia. (**A**) JN-170 pharmacokinetics in male *Pah^enu2^* mice after a single oral dose (*n* = 6). (**B**) Changes in 12 hour urine amino acid excretion in male *Pah^enu2^* mice treated with JN-170 (*n* = 4–6 per dose) or vehicle (veh, *n* = 24) or in mice with heterozygous or homozygous loss of *Slc6a19* (data adapted from (14)). Data are displayed as log FC. (**C**) 12-hour urine Phe excretion in mice treated with vehicle (*n* = 24) or JN-170 (*n* = 6); 1-way ANOVA with Tukey’s post hoc analysis. (**D**) Plasma Phe in male *Pah^enu2^* mice treated with vehicle (*n* = 24) or 50, 100, 200, or 250 mg/kg JN-170 (*n* = 6); 2-way ANOVA with Dunnett’s post hoc analysis to compare vehicle versus JN-170 groups (statistical analysis shown for comparison of vehicle to 250 mg/kg JN-170). (**E**–**G**) Plasma Phe in male *Pah^enu2^* mice fed defined amino acid diets to modulate basal plasma Phe levels. Mice were fed 0.75% Phe (**E**, *n* = 17–22), 0.45% Phe (**F**, *n* = 4–9) or 0.225% Phe (**G**, *n* = 11–17) for 2 weeks prior to administration of a single 200 mg/kg dose of JN-170 or vehicle; 2-way ANOVA with Šidák’s post hoc analysis.**P < 0.01, ***P < 0.001, ****P < 0.0001; All data represent mean ± SD.

In contrast to series 1, series 2 exemplars were more potent inhibitors of mouse SLC6A19 compared with the human ortholog (Figure 1C). The series 2 analog JN-170 (Figure 1E) inhibited mouse SLC6A19 with an IC_50_ of 97 nM (95% CI 73-129 nM) but was > 10-fold less potent against human SLC6A19 (IC_50_ = 1.25 μM, 95% CI 0.68–2.82 μM) (Figure 1F). The potency of JN-170 against mouse SLC6A19, combined with its favorable pharmacokinetic profile (Figure 2A and Figure 3A), made it a suitable tool molecule to investigate the effects of SLC6A19 inhibition on neutral amino acid liberation via urinary excretion in vivo.

### Inhibition of SLC6A19 causes aminoaciduria and lowers plasma Phe in a PKU mouse model.

Previous studies have shown that genetic loss of *Slc6a19* in mice recapitulates the phenotype observed in Hartnup disorder, characterized by increased urinary excretion of amino acids, including Phe, without causing a pathological decrease in plasma amino acid levels (20, 21). In the *Pah^enu2^* mouse model of PKU (22), homozygous knockout of *Slc6a19* likewise increased urinary excretion of neutral amino acids and lowered highly elevated plasma Phe by approximately 70% while plasma levels of other amino acids remained largely unaffected (14).

We first sought to determine whether pharmacological inhibition of SLC6A19 phenocopies the effects of genetic loss-of-function. To this end, WT C57Bl/6J mice were orally administered a single dose of JN-170 at levels ranging from 50 to 250 mg/kg to achieve sufficient exposure given the compound’s limited oral bioavailability of approximately 5% (Figure 2A). JN-170 caused a dose-dependent increase in urinary amino acid excretion (2-way ANOVA main effect of treatment *P* < 0.0001). At the highest dose (250 mg/kg), the largest increases were observed for Gln (84-fold; vehicle [veh]: 64 ± 13 μg, JN-170: 5,405 ± 1270 μg), His (32-fold; veh: 14 ± 3 μg, JN-170: 436 ± 100 μg), and Thr (29-fold; veh: 27 ± 5 μg, JN-170: 785 ± 187 μg), while the effects on urinary Pro were negligible (veh: 7 ± 2 μg, JN-170: 17 ± 4 μg) (Figure 2B), in line with observations in *Slc6a19*-KO animals and the substrate preference of the transporter (14).

We then explored the pharmacodynamic effects of JN-170 on urine neutral amino acid excretion and plasma Phe in the *Pah^enu2^* mouse. A dose of 50 mg/kg afforded coverage above the in vitro IC_50_ for approximately 8 hours and IC_75_ for approximately 3 hours, while the highest doses (≥ 200 mg/kg) afforded extensive coverage above the in vitro IC_90_ (> 12 hours) (Figure 3A). Similar to WT mice, a dose-dependent increase in urinary neutral amino acids was observed in *Pah^enu2^* mice following a single dose of JN-170 (Figure 3B and Supplemental Figure 3). At doses of ≥ 200 mg/kg, affording JN-170 exposure above the in vitro IC_90_ for the duration of the 12-hour urine collection, urinary Phe excretion was increased > 40-fold (veh: 96 ± 31 μg, 200 mg/kg: 4,170 ± 1505 μg), comparable with the approximately 50-fold increase observed with homozygous loss of *Slc6a19* (Figure 3C) (14). At comparable drug exposure levels (0–3 hours following a 200 mg/kg dose of JN-170, Supplemental Figure 4A), WT and *Pah^enu2^* mice excreted comparable amounts of glutamine (WT: 1,375 ± 362 μg, *Pah^enu2^*: 1,543 ± 606 μg; Supplemental Figure 4B). In contrast, the urinary excretion of Phe was approximately 60-fold higher in *Pah^enu2^* mice (2,115 ± 608 μg) compared with WT mice (37 ± 28 μg), suggesting that the elevated plasma Phe concentration in *Pah^enu2^* mice promotes higher levels of urinary Phe excretion (Supplemental Figure 4B).

To validate the hypothesis that pharmacological inhibition of SLC6A19 is sufficient to reduce the pathological blood Phe levels in the *Pah^enu2^* mouse model of PKU, plasma samples were analyzed by LC-MS/MS. While plasma Phe levels in *Pah^enu2^* mice display circadian fluctuations (Figure 3D) (23), JN-170 significantly drove reductions in plasma Phe in an exposure-related manner (Figure 3D). At the 50 mg/kg dose, plasma Phe was 45% lower 3 hours after dosing (984 ± 135 μM) compared with predose baseline (1,811 ± 255 μM) versus a 3% decrease following treatment with vehicle (predose: 1,566 ± 311 μM, 3 hours: 1,454 ± 192 μM, (*P* = 0.0003). By 12 hours after the dose, no significant difference in plasma Phe was observed between 50 mg/kg JN-170 (1,119 ± 124 μM, 34% decrease from baseline) and vehicle treatment (1,280 ± 152 μM, 22% decrease from baseline, *P* = 0.647). In contrast, a 250 mg/kg dose produced a 54% reduction in plasma Phe at 3 hours (764 ± 43 μM compared with predose Phe of 1,686 ± 205 μM) and the effect persisted to 12 hours after the dose (648 ± 106 μM, a 61% reduction versus a 22% reduction for vehicle control, *P* < 0.0001) (Figure 3D), driven by continuous plasma exposure of JN-170 above the in vitro IC_90_ (Figure 3A). Taken together, these data demonstrate that plasma exposures of JN-170 that afford target coverage above the IC_75_ are sufficient to lower plasma Phe levels by > 40%, while an approximately 60% decrease in plasma Phe is achieved with JN-170 exposures that afforded target coverage above the in vitro IC_90_.

To investigate whether SLC6A19 blockade may be efficacious in less severe forms of PKU, *Pah^enu2^* mice were maintained on defined amino acid diets with variable Phe contents (Figure 3, E–G). After 2 weeks on diet, mice fed 0.75% Phe had mean plasma Phe levels > 1,200 μM, mimicking a classic PKU phenotype (Figure 3E) while mice fed 0.45% Phe or 0.225% Phe diets had mean basal plasma Phe levels mimicking moderate PKU (> 900 μM but < 1,200 μM, Figure 3F) or hyperphenylalaninemia (> 360 μM but < 600 μM, Figure 3G), respectively. Plasma Phe levels were significantly lowered in *Pah^enu2^* mice treated with a single 200 mg/kg dose of JN-170 compared with vehicle on all 3 diets. Three hours after dose, mean plasma Phe in mice treated with JN-170 was 59%, 61%, and 47% lower than in mice treated with vehicle on 0.75% (veh: 1,502 ± 485 μM; JN-170: 621 ± 141 μM), 0.45% (veh: 1,063 ± 194 μM; JN-170: 416 ± 117 μM) and 0.225% (veh: 579 ± 205 μM; JN-170: 309 ± 108 μM) Phe diets, respectively. Taken together, these data suggest that pharmacological inhibition of SLC6A19 may be efficacious in PKU patients irrespective of their starting plasma Phe levels.

### JNT-517 is safe and well tolerated in healthy volunteers in single and multiple ascending dose studies.

**Figure 4:**
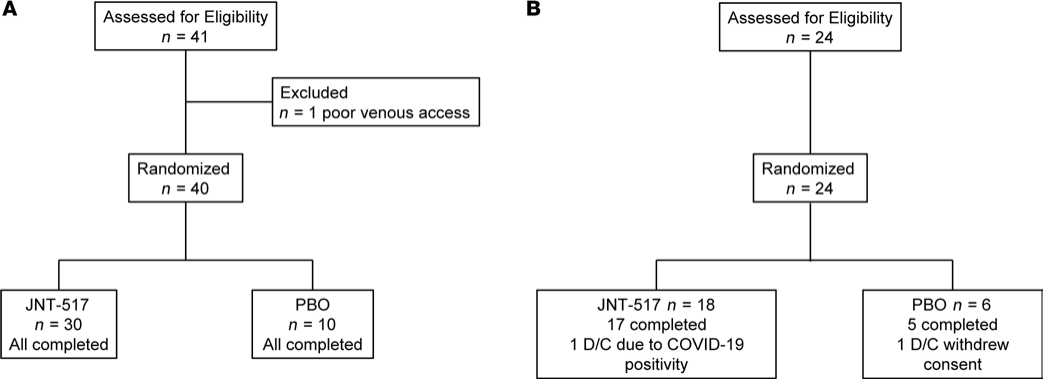
CONSORT diagram of patient disposition. (**A**) SAD cohort, (**B**) MAD cohort. D/C discontinued, PBO, placebo control.

We conducted a first in human (FIH), randomized, double-blind, placebo-controlled study to assess the safety, pharmacokinetics and pharmacodynamics of JNT-517 in healthy participants. Cohorts of 8 participants each (6:2 active versus placebo) were administered JNT-517 at single doses ranging from 10–170 mg and 14 day multiple-ascending doses of 25 mg twice daily (BID), 75 mg BID, or 150 mg once daily (QD) (Figure 4, A and B, and Supplemental Figure 5). Baseline characteristics were generally similar across the cohorts (Supplemental Table 1). Treatment with JNT-517 was safe and well tolerated. There were no serious adverse events or deaths reported in any part of the study. There were no clinically relevant drug-related changes in laboratories, ECGs, or vital signs and no dose-related trends in any part of the study. In the single ascending dose (SAD) cohorts, incidence of adverse events (AE) was 23.3% (7 of 30) with JNT-517 treatment versus 30.0% (3 of 10) with placebo (Table 1). The most common adverse event was headache (*n* = 2, 1 participant who received JNT-517, 10 and 50 mg each). In the multiple ascending dose (MAD) cohorts, the incidence of AE was 61.1% (11 of 18) with JNT-517 treatment versus 83.3% (5 of 6) with placebo (Table 2). Headache (all mild events, *n* = 7, 3 participants received JNT-517, 25 mg BID; 1 participant received JNT-517, 75 mg BID; 2 participants received 150 mg QD; 1 participant received placebo) was the most frequently reported treatment emergent adverse event (TEAE).

**Figure 5:**
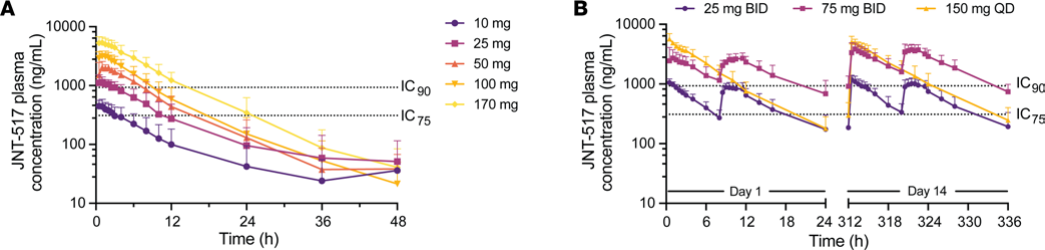
JNT-517 shows dose-proportional plasma exposure in single and multiple ascending doses in healthy human volunteers. (**A**) Pharmacokinetics of JNT-517 in plasma in SAD cohorts. (**B**) Day 1 and Day 14 pharmacokinetics of JNT-517 in plasma in MAD cohorts. Values represent mean ± SD for *n* = 5–6 individuals dosed with active compound per cohort. In vitro IC_50_ and IC_75_ values for JNT-517 adjusted for plasma protein binding indicated. BID, twice a day, QD, once daily.

JNT-517 demonstrated rapid absorption following oral administration and generally dose-proportional linear exposures with low variability, which were maintained through up to 2 weeks of dosing. In the SAD cohorts, JNT-517 total exposure (AUC) and maximal plasma concentration (Cmax) were dose-proportional up to 50 mg and slightly less than dose-proportional at the 100 and 170 mg doses (Figure 5A and Supplemental Table 2). A single 50 mg dose provided exposures above the in vitro IC_75_ for > 12 hours and above the in vitro IC_90_ for > 6 hours. At the highest dose of 170 mg, target coverage exceeded the IC_75_ for approximately 24 hours and the IC_90_ for > 12 hours (Figure 5A). In the MAD cohorts, no accumulation was observed between days 1 and 14 at the dose levels tested (Figure 5B). In the 150 mg QD cohort, plasma exposures exceeded the in vitro IC_75_ and IC_90_ for approximately 24 hours and approximately 12 hours, respectively, while dosing at 75 mg BID afforded exposures approaching or exceeding the IC_90_ for approximately 24 hours.

### JNT-517 increases neutral amino acids in the urine of healthy volunteers in single and multiple ascending dose studies.

**Figure 6:**
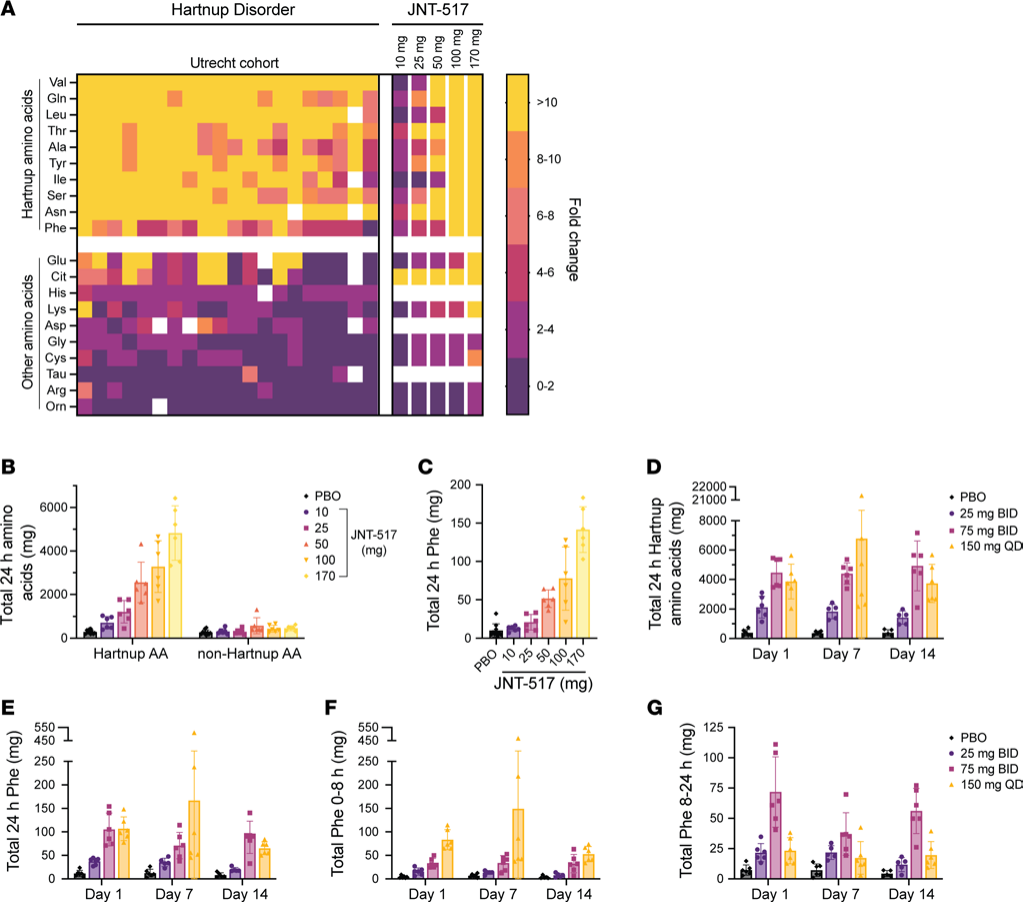
JNT-517 increases urinary neutral amino acid excretion in healthy human volunteers, reproducing the Hartnup aminoaciduria signature caused by genetic loss of *SLC6A19* function. Pharmacodynamics of JNT-517 in SAD (**A**–**C**) and MAD (**D**–**G**) cohorts. Urine amino acids excreted over various time periods (0–24 hours, 0–8 h, 8-24 hours) are shown. (**A**) Fold changes of urinary amino acid concentrations (in mmol/mol creatinine) following a single dose of JNT-517, calculated relative to predose baseline in samples collected over 8 hours following compound administration from 10–170 mg. FCs represent average FC for all participants at each dose level. Hartnup amino acid data were adapted from the Utrecht cohort reported by Haijes et al. (11). (**B**) Total 24 hour Hartnup and non-Hartnup amino acids (in mg) excreted following single ascending doses (10–170 mg) of JNT-517. (**C**) Total amount of urinary Phe excreted over 24 hours following single ascending doses (10–170 mg) of JNT-517. (**D**) Total 24 hour Hartnup amino acids excreted on days 1, 7, and 14 in MAD cohorts (dose levels 25 mg BID, 75 mg BID, 150 mg QD). (**E**–**G**) Total urinary Phe excreted on days 1, 7, and 14 in multiple ascending dose cohorts 0–24 hours (**E**), 0–8 hours (**F**) and 8–24 hours (**G**) after dose. Hartnup AAs are the sum of Ala, Asn, Gln, Ile, Leu, Phe, Ser, Thr, Tyr, Val. Non-Hartnup AAs are the sum of Arg, Cys, Glu, Gly, Lys, Pro. Values represent mean ± SD for *n* = 5–6 individuals dosed with placebo or active compound per cohort. BID, twice daily; PBO, placebo; QD, once daily

The pharmacodynamic effects of JNT-517 were assessed by measuring urine amino acids. Amino acids were grouped as either Hartnup amino acids (Gln, Phe, Ala, Asn, Ile, Leu, Ser, Thr, Tyr, and Val, preferred substrates of SLC6A19), or non-Hartnup amino acids. In the SAD cohorts, a single dose of JNT-517 was sufficient to achieve a dose-dependent increase in the amount of individual (Figure 6A, 8-hour collection) and total (Figure 6B, 24-hour collection) Hartnup amino acids excreted in the urine. The total amount of non-Hartnup amino acids remained largely unchanged at all dose levels (Figure 6, A and B). These data recapitulate the aminoaciduria pattern observed with genetic loss of *SLC6A19* in a cohort of individuals with Hartnup disorder at Utrecht University (11) (Figure 6A). In the highest dose group (170 mg), 142 ± 30 mg Phe was excreted over a 24 hour period, compared with 10 ± 9 mg in the placebo group (approximately 14-fold increase) (Figure 6C). Broadly, the amount of amino acid excretion correlated with the plasma amino acid concentration. Glutamine, the amino acid with the highest plasma concentration, was also the most abundant amino acid excreted (1,904 ± 541 mg in the 170 mg JNT-517 group compared with 99 ± 34 mg in the placebo group) (Supplemental Figure 6).

In the MAD study, the total amount of Hartnup amino acids excreted over 24 hours was dose dependent (Figure 6D). No signs of tachyphylaxis were observed after 14 days of dosing in any dose group. Comparable amounts of total Hartnup amino acids (Figure 6D), including Phe (Figure 6E) were excreted in the 75 mg BID and 150 mg QD dosing groups over a 24 hour period (24 hour day 14 Phe excretion: 89 ± 34 mg versus 72 ± 18 mg in the 75 mg BID and 150 mg QD cohorts, respectively). The onset of the pharmacodynamic effect was rapid, with elevations in Hartnup amino acids observed within the first 8 hours after dose (Figure 6F). Pharmacodynamic differences between the 75 mg BID and 150 mg QD groups emerged when 0–8 hours (Figure 6F) and 8–24 hours (Figure 6G) urine collection intervals were analyzed separately. In the 150 mg QD cohort, the amount of Phe excreted from 0–8 hours was higher than in the 75 mg BID group (day 14: 32 ± 20 mg compared with 53 ± 16 mg for 75 mg BID and 150 mg QD cohorts, respectively), in line with the higher JNT-517 exposure afforded during that time. In contrast, from 8–24 hours, the amount of urine Phe excreted was higher for the 75 mg BID dose (day 14: 56 ± 19 mg) than for the 150 mg QD dose (day 14: 20 ± 11 mg). This is in line with the decrease in exposure below the IC_90_ after approximately 12 hours for the 150 mg QD dose while exposure remained near continuously above the IC_90_ for the 75 mg BID dose. Plasma amino acid levels following JNT-517 dosing remained largely within normal ranges with few transient decreases measured for individual amino acids, which were reversible and mainly occurred in participants with low plasma amino acid levels prior to compound administration (Supplemental Table 3).

## Discussion

Here, we demonstrate for what is, to our knowledge, the first time that pharmacological inhibition of SLC6A19 increases the urinary excretion of neutral amino acids, including Phe, and reduces plasma Phe levels in a mouse model of PKU. In addition, we have described initial clinical proof of mechanism from a phase 1 healthy volunteer study of JNT-517, a first-in-class inhibitor of SLC6A19, which resulted in profound aminoaciduria at pharmacologically relevant doses that were safe and well tolerated. Taken together, these results indicate that small molecule inhibition of SLC6A19 may provide new treatment opportunities for PKU as well as other inborn errors of metabolism characterized by the toxic accumulation of neutral amino acids.

Two chemical series identified using RAPID, a high throughput reactive affinity probe displacement screen, displayed functional inhibition of SLC6A19. Optimization of 1 series yielded JN-170, a potent inhibitor of mouse SLC6A19. WT and *Pah^enu2^* mice, a widely used mouse model of PKU, displayed dose-dependent aminoaciduria upon treatment with JN-170, including increased urinary excretion of Phe, confirming that pharmacological inhibition of SLC6A19 phenocopies the loss-of-function phenotype observed in *Slc6a19*-KO mice as well as in humans with Hartnup disorder, which results from mutations in *SLC6A19*. Furthermore, in *Pah^enu2^* mice, JN-170 lowered plasma Phe in a dose-dependent manner, suggesting that a small molecule SLC6A19 inhibitor may provide an effective therapeutic approach to liberate excess plasma Phe. In this preclinical disease model, exposures above the in vitro IC_75_ were sufficient to elicit a > 40% reduction in plasma Phe, while exposures > IC_90_ maximally increased urine Phe and other neutral amino acids and achieved a reduction in plasma Phe of > 60%. These data are in line with genetic data showing that heterozygous loss of *Slc6a19* is insufficient to lower plasma Phe in *Pah^enu2^* mice and suggest that steady-state exposures above the IC_90_ may be desired to exercise the maximal pharmacological effect on liberation of excess Phe in a clinical PKU setting.

While both genetic loss of *Slc6a19* function (14) and JN-170 were shown to lower plasma Phe in *Pah^enu2^* mice with highly elevated baseline Phe levels (> 1,200 μM), interindividual variability in plasma Phe is high in patients with PKU, and 38% of adult patients with PKU have plasma Phe levels that are elevated but are under 1,200 μM (24). In order to investigate whether SLC6A19 inhibition may be efficacious in patient populations with nonclassic PKU, dietary Phe intake was modulated in *Pah^enu2^* mice to achieve varying severities of baseline plasma Phe. Results demonstrated that JN-170 was effective at lowering plasma Phe even in *Pah^enu2^* mice on a low Phe diet, mimicking a hyperphenylalaninemia phenotype (mean Phe < 600 μM), compared with vehicle control. These data suggest that PKU patients with hyperphenylalaninemia rather than classic PKU may likewise benefit from pharmacological SLC6A19 inhibition.

JNT-517, a potent and selective first-in-class inhibitor of human SLC6A19, was progressed into phase 1 clinical evaluation. JNT-517 was found to be safe and well tolerated in single and multiple ascending dose studies in healthy volunteers. JNT-517 caused a dose-dependent increase in urinary Hartnup amino acids in both SAD and MAD cohorts, reproducing the aminoaciduria signature observed in Hartnup disorder resulting from loss-of-function mutations in *SLC6A19* and corroborating the pharmacology of SLC6A19 inhibition in mice. The amount of each excreted amino acid broadly correlated with its corresponding plasma concentration. Preclinically, *Pah^enu2^* mice dosed with SLC6A19 inhibitor excreted approximately 60 × more Phe than WT mice. Taken together, these data suggest that in patients with PKU, inhibition of SLC6A19 may effect a higher degree of urinary Phe excretion than that observed in healthy volunteers, providing a stronger drive to lower the pathologically elevated levels of this amino acid in blood. This is analogous to the enhanced glucosuria observed with inhibition of the renal glucose transporter SGLT2 in Type 2 diabetes (25, 26). Furthermore, given the transporter’s broad substrate specificity, JNT-517 may provide therapeutic benefits for other inborn errors of metabolism characterized by the toxic accumulation of SLC6A19 transport substrates or their metabolites.

Plasma amino acid levels of study volunteers in the MAD cohorts remained largely within the normal range after 14 days of treatment with JNT-517, despite the increased loss of amino acids via urinary excretion. This is in line with observations in individuals with Hartnup disorder, who generally maintain amino acid homeostasis and are asymptomatic when provided with a protein-rich diet (12, 13), as well in *Slc6a19*-KO mice, which likewise maintain normal levels of plasma amino acids through metabolic adaptations that include limiting the use of amino acids as fuel or reducing muscle protein turnover and degradation (14, 20, 21, 27). Symptomatic cases of Hartnup disorder, which can present with pellagra-like skin lesions and neurological manifestations, are thought to be caused by a deficiency in niacin, which is synthesized from its precursor tryptophan (12, 13). While symptoms tend to present more frequently in individuals who are on a severely restricted protein diet, there were no reports of clinical manifestations of Hartnup-like symptoms in the healthy volunteer SAD or MAD cohorts. Two cases of plasma tyrosine levels below normal were noted in the 150 mg QD MAD cohort that were transient, did not require intervention, and were not associated with adverse events of amino acid reduction. Tyrosine is synthesized from Phe and may be low in patients with PKU due to the impairment of Phe metabolism and protein-restricted diet. In future long-term studies, plasma amino acid levels, including tyrosine and tryptophan, and clinical presentations that may suggest reduction of tryptophan or niacin, will be monitored in patients with PKU to assess the long-term effects of SLC6A19 inhibition and resulting aminoaciduria.

The metabolic adaptations that maintain plasma amino acid levels in mice with genetic loss of *Slc6a19* despite broad aminoaciduria do not extend to the toxic levels of Phe in the *Pah^enu2^* mouse model of PKU; there, genetic loss of *Slc6a19* selectively lowered the highly elevated plasma Phe levels while other plasma amino acids were largely unaffected (14). This selective lowering of Phe in the PKU context may be explained by allosteric regulation of enzymes responsible for amino acid catabolism. Under conditions of enhanced urinary neutral amino acid excretion, catabolism of these amino acids can be reduced to maintain plasma homeostasis, with the exception of Phe, given that functional deficiency of the Phe-metabolizing enzyme PAH is the underlying cause of PKU in the first place. The homeostatic drive to maintain plasma amino acids was evident in healthy human volunteers treated with JNT-517, which largely maintained plasma amino acid levels in the normal range despite urinary loss of amino acids. Clinical evaluation of JNT-517 in patients with PKU will demonstrate whether pharmacological SLC6A19 inhibition can selectively lower pathologically elevated levels of plasma Phe without affecting the homeostatic control of nonpathological amino acids.

The cornerstone of treatment for PKU is a life-long severely Phe-restricted diet. The majority of patients struggle to adhere to the diet and are unable to maintain plasma Phe levels within limits articulated in treatment guidelines (< 360 μM in the US; < 360 μM in Europe for children < 12 years and < 600 μM for 12+ years). Currently approved interventional treatments provide limited therapeutic benefit as they either only work in patients with residual enzyme activity or are associated with substantial adverse events that have limited broader utilization. Here, we provide evidence that oral administration of a small molecule inhibitor of the amino acid transporter SLC6A19 can block renal neutral amino acid reabsorption and may provide a compelling therapeutic approach for PKU and related disorders of amino acid metabolism. In human studies, JNT-517 was safe and well tolerated and delivered profound aminoaciduria. Following completion of healthy volunteer studies, JNT-517 has progressed into clinical evaluation in patients with PKU.

## Methods

### Sex as a biological variable.

Mouse studies in C57Bl/6J and *Pah^enu2^* mice used male mice only to minimize variability. Human clinical studies involved both male and female volunteers, and numbers of both sexes are detailed in the baseline characteristics of the study cohorts.

### Generation and maintenance of stable SLC6A19-expressing cell lines.

Flp-In T-REx 293 cells (Thermo Fisher Scientific) were engineered to coexpress C-terminally V5-tagged human or mouse SLC6A19 under the control of a tetracycline-inducible promoter and human or mouse myc-DDK-tagged TMEM27 under the control of a constitutively active promoter. Cells were maintained in DMEM/F12 medium supplemented with 10% FBS, 100 units/mL penicillin, 100 μg/mL streptomycin, 200 μg/mL hygromycin, 10 μg/mL blasticidin, and 300 μg/mL geneticin (all from Thermo Fisher Scientific) at 37°C and 5% CO_2_.

### Isoleucine transport assay.

Flp-In T-REx 293 cells stably coexpressing human or mouse SLC6A19 and TMEM27 were plated in 384-well plates in the presence of 1 μg/mL tetracyclin (Sigma-Aldrich) to induce the expression of SLC6A19. After 24 hours, cells were washed with live cell imaging solution (LCIS, Thermo Fisher Scientific) before JN-170 or JNT-517 were added to cells in 10-point concentration response curves in 0.5% DMSO in Krebs buffer (140 mM NaCl, 4.7 mM KCl, 2.5 mM CaCl_2_, 1.2 mM MgCl_2_, 11 mM HEPES, 10 mM glucose, pH 7.4). Cells were incubated with compounds for 1 hour at room temperature before 1 mM ^13^C_6_,^15^N_1_-labeled L-isoleucine (Cambridge Isotope Laboratories) was added as a transport substrate. After 20 minutes incubation at room temperature, cells were washed and lysed in H_2_O with 15 μM internal standard D-Leucine-D_10_ (CDN Isotopes) for 1–2 hours. A standard curve of ^13^C_6_,^15^N_1_-L-isoleucine was added to each plate following cell lysis. Plates were centrifuged at 3,700*g* to remove cell debris, and supernatant was diluted in 2 steps to a final 1:10 dilution in acetonitrile/0.1% formic acid. Sample desalting was performed on a high throughput RapidFire-365 (Agilent) using a hydrophilic interaction liquid chromatography (HILIC) column. A quantitative time-of-flight (Q-ToF) mass spectrometer (Agilent) was used to detect analytes. Agilent MassHunter quantitative analysis for time of flight was used as the integration software for measuring area under the peak for both ^13^C_6_,^15^N_1_-L-isoleucine and D-Leucine-D_10_ internal standard.

### Isolation of human epithelial cells from postmortem small intestine.

Human postmortem intestines from 3 male donors (ages 23–36 years) were provided by AnaBios. Following trimming of excess fat, the distal part of the jejunum was dissected into tissue sections of approximately 100 cm^2^ and further processed on ice. These sections contained an estimated approximately 250 million epithelial cells (28, 29). Epithelial cells were scraped off each tissue section with a tissue scraper and collected in ice-cold Hank’s balanced salt solution (HBSS). Following centrifuging for 5 min at 300*g* at 4°C, the supernatant was aspirated and the cell pellet was washed 3–6 times by resuspending in ice-cold HBSS (Thermo Fisher Scientific). After the last washing step, the cell pellet was resuspended in ice-cold advanced DMEM/F12 medium and cells were filtered through a 500 mm cell strainer. Cells were centrifuged for 5 minutes at 300*g* at 4°C and resuspended in sodium-free Krebs buffer (140 mM choline chloride, 4.7 mM KCl, 2.5 mM CaCl_2_, 1.2 mM MgCl_2_, 11 mM HEPES, 10 mM glucose, pH 7.4 [all from Sigma-Aldrich]) and kept on ice until the start of the experiment.

### SLC6A19 glutamine transport assay in intestinal epithelial cells.

Human postmortem epithelial cells isolated from small intestine were centrifuged for 5 minutes at 300*g* and 4°C and resuspended in Krebs buffer (140 mM sodium chloride, 4.7 mM KCl, 2.5 mM CaCl_2_, 1.2 mM MgCl_2_, 11 mM HEPES, 10 mM glucose, pH 7.4) at a density of 7.5 × 10^6^ cells/mL. The cell suspension was transferred to V-bottom plates (750,000 cells per well) and JNT-517 was added in 10-point quadruplicate dose response using a Tecan D300e digital compound dispenser. Cell plates were incubated at room temperature for 60 minutes on a shaker at 400 rpm. Following compound incubation, L-glutamine, spiked with 2,3,4-^3^H–labeled L-glutamine, was added to the cells at a final concentration of 430 μM. Cells were incubated with substrate for 20 minutes at room temperature on a shaker at 400 rpm. Following substrate incubation, cells were centrifuged at 2,500*g* for 1 min at 4°C, washed once with ice-cold LCIS, and centrifuged. The supernatant was removed, and cells were lysed in water for a minimum of 30 minutes. Cell lysates were then transferred into scintillation fluid in a 96-well isoplate (Millipore) and incubated at room temperature overnight on a shaker. The uptake of 2,3,4-^3^H–labeled L-glutamine was measured on a Microbeta^2^ microplate counter (PerkinElmer). 

### JN-170 dosing and biospecimen collection.

Male homozygous BTBR-Pah<enu2>/J (*Pah^enu2^*) aged 10–13 weeks were purchased from Jackson Laboratory (no. 002232). Immediately before dosing, each animal’s bladder was manually emptied and a plasma sample was drawn. Animals were dosed by oral gavage with vehicle (10% DMSO, 20% 2-hydroxypropyl-β-cyclodextrin [Sigma-Aldrich]) followed by a 1-week washout prior to dosing with JN-170 at doses ranging from 50 to 250 mg/kg. Following dosing with vehicle or JN-170, animals were individually placed in metabolic cages to collect urine samples. After collection of each urine sample, cages were rinsed with ultra-pure water to recover residual analytes. Blood samples were collected predose as well as 1, 3, 8, 12, and 24 hours after dosing with vehicle or JN-170 into tubes with EDTA as a coagulant, and plasma was separated by centrifuging samples at 2,200*g* for 5 minutes at 4°C. Throughout the study, mice had access to food and water ad libitum. Plasma and urine samples were stored at –80°C until processing.Ten-week-old male WT C57Bl/6J animals (Si Bei Fu Laboratory Animal Technology Co. Ltd.) were dosed either with vehicle or 50–250 mg/kg JN-170 by oral gavage and blood and urine samples were collected as described. All animals had unrestricted access to food and water during the study; C57Bl/6J were kept on rodent maintenance diet from Beijing Keao Xieli, *Pah^enu2^* mice were kept on Teklad diet no. T.2018.

To modulate baseline plasma Phe levels, 8–13-week-old male *Pah^enu2^* mice were maintained on isocaloric amino acid–defined diets with either 7.5 g/kg (0.75%, Teklad T.2018.15), 4.5 g/kg (0.45%, Teklad custom formulation), or 2.25 g/kg (0.225%, Teklad custom formulation) L-Phe for 2 weeks before a single dose administration of 200 mg/kg JN-170 or vehicle. Animals had unrestricted access to food and water throughout the study. Serial plasma samples were taken predose as well as 1, 3, 8, 24 and 48 hours after the dose. For all studies, mice were dosed at 7:00 a.m. ± 30 minutes to synchronize with circadian rhythm.

### Mouse plasma JN-170 and Phe analysis.

Plasma sample analyses were carried out by liquid chromatography tandem mass spectrometry (LC-MS/MS). Plasma samples were mixed 1:1 with carbamazepine or ^13^C_6_-Phe as internal standards for JN-170 and Phe, respectively. Plasma proteins were precipitated by adding 4 volumes of acetonitrile (ACN) and 0.1% formic acid (FA) followed by centrifugation for 5 minutes at 2,100*g*. Supernatants were diluted 1:1 in ACN and 0.1% FA. For JN-170, a Waters HSS T3 reversed phase column was used for separation using a binary gradient of 0.1% FA in water (mobile phase A) to 0.1% in ACN (mobile phase B). At a flow rate of 0.8 mL/min, the mobile phase gradient increased from an initial 25% phase B to 95% phase B over 30 seconds. After, 1.5 minutes, the gradient was returned to 25% mobile phase B. For detection of Phe, an Acquity UPLC ethylene bridged hybrid (BEH) amide column was used, with a binary gradient of 10 mM ammonium acetate in 95:5:1 H_2_O:ACN:FA (mobile phase A) to 10 mM ammonium acetate in 95:5:1 ACN:H_2_O:FA (mobile phase B). At a flow rate of 0.5 mL/min, the mobile phase gradient changed from an initial 100% phase B to 50% phase B over 2.1 minutes. It was returned to 100% phase B after 4 minutes. Analytes were detected using a Sciex API-5500 in multiple reaction monitoring mode using an electrospray ionization source in the positive ion mode with the following ion transitions: m/z 435.2/84.1 (JN-170), 237.1/194.1 (carbamazepine), 166.1/120.1 (Phe), 172.2/126.2 for (^13^C_6_-Phe). Analyte concentrations were calculated with a 1/×^2^ linear regression over a concentration range of 1 or 2 to 5,000 ng/mL for JN-170 and 0.05–100 ng/mL for Phe.

### Mouse urine amino acid analysis.

Urine and cage-wash samples were vortexed and centrifuged at 3,700*g* for 5 minutes to remove any solid precipitate. Neat samples and samples diluted 1:10 in water were further diluted 1:10 in crash solution (75% acetonitrile 25% methanol, 0.1% formic acid, 5 mM heavy labeled amino acids) to precipitate proteins. Samples were centrifuged at 3,700*g* for 5 minutes before the supernatant was transferred into an injection plate. Urine amino acid analysis was carried out using a quadrupole time of flight (QToF) LC-mass spectrometry (QToF-LC-MS) bioanalytical method. A standard curve of 1 mM down to 0.06 μM was used as the calibration curve to which samples were quantified. The amino acid concentrations were calculated with a 1/×2 linear regression over a concentration range of 60 nM to 1,000 μM of the individual amino acids. An Agilent QToF 6545 was operated in an accurate mass full scan range of 100–1,700 m/z positive ions formed by electrospray ionization with a normal phase HILIC chromatographic method. An Agilent InfinityLab Poroshell 120 HILIC-Z, 2.1 × 150 mm, 2.7 μm was used as the analytical column with 10 mM Ammonium Acetate in 0.1% FA as mobile phase A and 10 mM Ammonium Acetate in 95% ACN, 0.1% FA as mobile phase B. At a flow rate of 0.25 mL/min, the binary gradient increased from an initial 100% phase B to 90% phase B over 3 minutes. After 1.5 minutes, the gradient ramped to 80% mobile phase B. At 5.6 minutes into the gradient, mobile phase B ramped from 60% to 0% and held for 2 minutes before returning to initial conditions at 7.5 minutes. The gradient was stopped at 10 minutes.Total urine amino acid amounts were calculated by multiplying urine amino acid concentrations by the urine volume and adding the amount of amino acid recovered in the cage wash, which was calculated by multiplying the cage wash amino acid concentration by the cage wash volume.

### FIH study design, objectives, and conduct.

Parts A and B of this phase 1, randomized, double-blind, placebo-controlled SAD and MAD oral dose study with JNT-517 were conducted in healthy volunteers. The primary objective for these studies was to assess the safety and tolerability of single and multiple doses of JNT-517 in healthy participants. The secondary objective for these studies was to assess the PK of single and multiple oral doses of JNT-517. In addition, the pharmacodynamic effects of JNT-517 in urine and plasma amino acid levels after single and multiple dosing were assessed as an exploratory objective.

Eligible participants included males and females 18 to 55 years of age, medically healthy with no clinically notable medical history, a BMI of 18–40 kg/m^2^ and total body weight > 50 kg (110 lbs), and who have been nonsmokers for at least 2 weeks prior to and throughout dosing.

In total, 40 participants who were healthy adult were randomized to receive JNT-517 (10–170 mg) or placebo (3:1 ratio) in 5 cohorts of 8 participants as SADs. A total of 24 healthy participants were similarly randomized to receive JNT-517 or placebo for 14 days in 3 cohorts of 8 participants in MADs of 25 mg and 75 mg twice daily or 150 mg once daily. Within each SAD cohort, a sentinel group of 2 participants were initially randomized to receive JNT-517 or placebo in a 1:1 ratio. Once safety was confirmed for these 2 sentinel participants over a 48-hour observation period, the remaining 6 participants in the same cohort were randomized 5:1 to receive JNT-517 or placebo. Both JNT-517 and placebo were administered as an on-site compounded suspension.

### Human safety, PK, and PD assessment.

In both SAD and MAD studies, safety and tolerability were assessed by AE monitoring, clinical laboratory tests (blood chemistries, hematology, coagulation, serology, and urinalysis), vital signs, 12-lead electrocardiograms, and physical examinations. Safety assessment and corresponding samples were collected on day-1 prior to dosing and days 1, 2, 3, and 8 for the SAD study or throughout the 14 days of dosing and day 21 for the MAD study.

The plasma pharmacokinetic profile of JNT-517 was measured using a qualified high-performance LC-MS assay. Blood samples for PK analysis were taken before dosing, and 0.5, 1, 1.5, 2, 2.5, 3, 4, 6, 8, 10, 12, 24, 36, 38, and 72 hours after dosing in the SAD study. For the MAD study, blood samples for PK analysis were taken before dosing, and 0.5, 1, 1.5, 2, 2.5, 3, 4, 6, 8, 10, 12, and 24 hours after dosing in the case of once-daily dosing or 0.5, 1, 1.5, 2, 2.5, 3, 4, 6, 8.5, 9 9.5, 10, 10.5, 11, 12, 14, 16, and 24 hours after dosing in the case of twice daily dosing. In addition, predose samples were collected throughout days 3–13. The resulting concentration-time data was evaluated using standard noncompartmental analysis methods, and PK parameters were calculated (Cmax, Tmax, T_1/2_, AUC_0-inf_, CL/F, Vd/F).

The effect of JNT-517 on plasma and urine AA levels was measured using an ultra-performance liquid chromatography mass spectrometry assay following 6-aminoquinolyl-N-hydroxysuccinimidyl carbamate (AQC) derivatization. For the SAD study, plasma samples were collected on day –1 and day 1 predose, as well as 24, 48, and 72 hours after dosing. A first morning void urine sample on Days –1 and 1 predose, as well as 2 pooled urine samples 0–8 hours after and 8–24 hours after dose were also collected following study drug administration. For the MAD study, plasma samples were collected on day –1, 1, 7, and 14 predose, as well as day 14, 24 hours after dosing. A first morning void urine samples on day –1, 1, 7, and 14 as well as 2 pooled urine samples 0–8 hours and 8–24 hours after dose on days 1, 7, and 14 were also collected following JNT-517 or placebo administration. Study participants in SAD and MAD cohorts were dosed in the morning following an overnight fast of at least 8 hours and were provided the following meals in relation to dosing times: breakfast after the morning dose, lunch 4 hours after morning dose, optional snack in the afternoon 8 hours after the morning dose and after the second dose for MAD cohorts dosed BID, dinner approximately 10 hours after the morning dose and at least 2 hours after the second dose for MAD cohorts dosed BID. Predose plasma amino acids were drawn after a minimum of 8 hours fasting overnight and after-dose levels were drawn after > 1.5 hours fasting. Resulting plasma and urine amino acid concentration measurements, as well a total amino acid excretion over a time period were summarized using appropriate descriptive statistics and compared with either predose levels or placebo-dose values. Tryptophan was not included in the amino acid panel due to analytical limitations.

### Statistics.

IC_50_ values for transport inhibition in cells overexpressing SLC6A19 and TMEM27 were calculated using a 4-parameter logistic regression in Collaborative Drug Discovery (CDD). Reported IC_50_ values are geometric means of 4–6 independent experiments. For postmortem intestinal transport assays, single outliers of quadruplicate replicates were excluded from the calculation if a signal value’s distance to the mean exceeded 35% of the signal average. The IC_50_ values of individual experiments were calculated with a 3- or 4-parameter logistic regression using GraphPad Prism 9. The reported IC_50_ value represents the geometric mean of *n* = 5 experiments from 3 individual donors. Statistical comparison of urinary Phe excretion in *Pah^enu2^* mice treated with vehicle or JN-170 was conducted using 1-way analysis of variance (ANOVA) with Tukey’s post hoc comparison. Statistical analysis of baseline-adjusted plasma Phe in mice treated with vehicle or JN-170 was determined by 2-way ANOVA with Dunnett’s post hoc comparison. All statistical analyses were performed using GraphPad Prism 9. For clinical studies, group sizes were not determined based on statistical power calculations. Statistical significance of pharmacodynamic results was not assessed. Values are reported as mean (urinary Phe excretion, T_1/2_, Cl/F), geometric mean (C_max_/D, AUC_0-inf_/D), or median (T_max_).

### Study approval.

The human volunteer study was conducted at a single clinical research unit in Australia and was approved by the Alfred Hospital Ethics Committee, The Alfred Hospital, Commercial Road, Prahan, Victoria 3181, Australia. The study was registered on the Australian New Zealand Clinical Trials Registry (ANZCTR) on 09/09/2022 with the registration number ACTRN12622001222730. The study complied with all relevant ethical regulations, and all participants in the study provided written informed consent. Mouse studies were approved by the Institutional Animal Care and Use Committees (IACUC) at Charles River Laboratories, Pharmaron, and Mispro.

### Data availability.

Data underlying graphs depicting in vitro and murine studies are provided in the Supporting Data Values file. Human volunteer PK and PD data may be provided upon request.

## Author contributions

HJW, TJR, MTL, and AV designed preclinical studies. HJW, DVK, CTB, SMC, RMB, and LG conducted experiments. HJW, DVK, HSB, and AV analyzed preclinical data. YRD oversaw the preparation of human intestinal epithelial cells. HJW, AV, GM, RH, NP, JLH, JCB, and DGB directed research. MTA and JEZ designed synthetic routes. HHW, TAV, AV, and JPT designed the clinical study. HHW, AV, and JPT participated in clinical assessment. TAV, JPT, and HHW provided oversight for the execution of the clinical study. HSB analyzed and interpreted the clinical PK data. FW supplied materials for the clinical study. HJW, AV, HHW, and JPT wrote the manuscript, and all authors reviewed the manuscript.

## Supplementary Material

Supplemental data

ICMJE disclosure forms

Supporting data values

## Figures and Tables

**Table 1:**
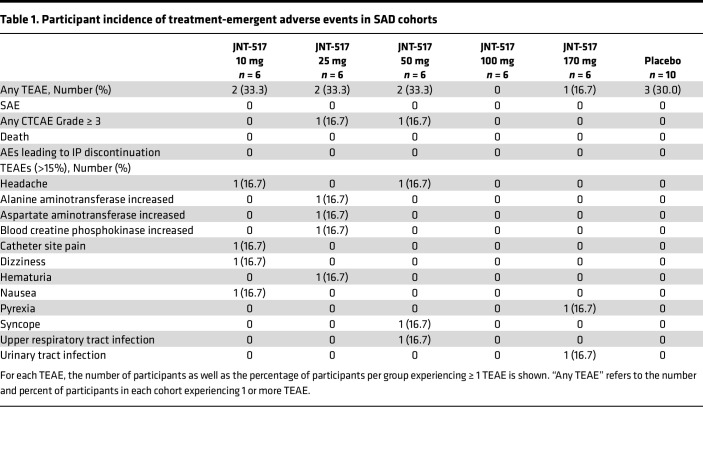
Participant incidence of treatment-emergent adverse events in SAD cohorts

**Table 2:**
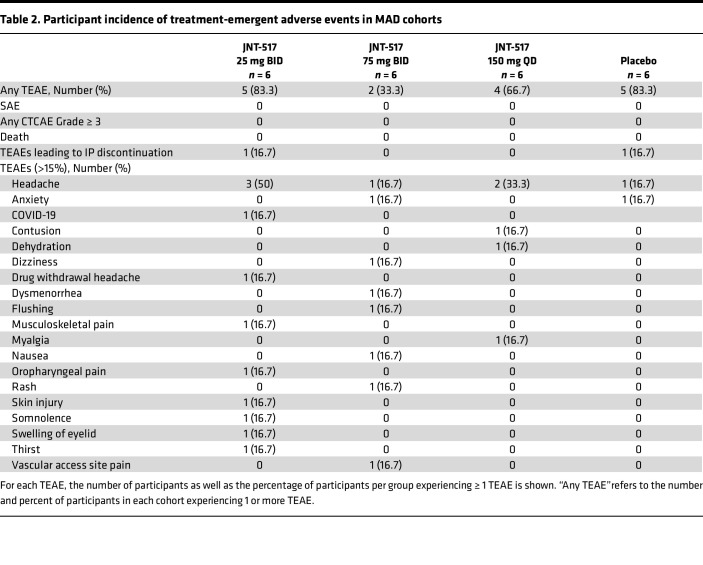
Participant incidence of treatment-emergent adverse events in MAD cohorts

## Version 1 11/08/2024

Electronic publication
