# Supplementary material for: SLC6A19 inhibition facilitates urinary neutral amino acid excretion and lowers plasma phenylalanine
Source: JCI Insight. 2024 Nov 8;9(21):e182876. doi: 10.1172/jci.insight.182876 (PMC11601558; doi:10.1172/jci.insight.182876)
Supplement: Supplemental data [file jciinsight-9-182876-s303.pdf]

Supplementary Materials and Methods

Cell culture

Flp-In T-Rex 293 cells (Thermo Fisher) were engineered to inducibly express either V5-tagged human SLC1A5, SLC6A8 or SLC7A5 co-expressed with myc-DDK-tagged SLC3A2. Cells expressing SLC1A5 or SLC6A8 were maintained in either DMEM or DMEM/F12 media supplemented with 10% FBS, 100 U/mL penicillin, 100 μg/mL streptomycin, 200 μg/mL hygromycin, and 10 μg/mL blasticidin. For cells expressing SLC7A5/SLC3A2, the media was additionally supplemented with 300 μg/mL geneticin. All cell lines were maintained at 37°C and 5% CO_2. For transport experiments, all cell lines were plated in poly-D-lysine-coated 96-well plates at a density of 35,000 cells/well. One day after plating, expression of SLC1A5, SLC6A8 or SLC7A5 was induced by adding tetracycline at a concentration of 1 μg/mL. Cells were induced for 24 h prior to transport assays.

SLC1A5 glutamine transport assay

Following a 24 h induction with tetracycline, cells were washed with LCIS before JNT-517 was added in 8-point concentration-response diluted in live cell imaging solution. D-serine was used as a positive control (32) at a concentration of 40 mM and 0.5% DMSO was used as a negative control. Cells were incubated for 20 min at room temperature and then placed on ice for an additional 5 minutes. Then, a mixture of unlabeled L-glutamine (Millipore Sigma) spiked with 4.5 μCi/mL 2,3,4-^{3}H-labeled L-glutamine (American Radiolabeled Chemicals) was added at a final concentration of 100 μM. After a 5 min incubation on ice, cells were washed once with ice-

cold LCIS and lysed in scintillation fluid (PerkinElmer). The uptake of radioactive substrate was measured on a Microbeta^2 microplate counter (PerkinElmer).

SLC6A8 creatine transport assay

Following a 24 h induction with tetracycline, cells were washed with PBS before JNT-517 was added in 8-point concentration-response diluted in DMEM. 3-guanidinopropionic acid was used as a positive control (33) at a concentration of 1 mM and 0.5% DMSO was used as a negative control. Cells were incubated in a cell culture incubator for 30 minutes at 37°C and 5% CO_2 before 15 mM D3-labeled creatine (CDN Isotopes) was added as a transport substrate. After a 15 min incubation at 37°C and 5% CO_2, cells were washed once with PBS. Cells were lysed in H_2O with 500 nM D5-labeled creatine (CDN isotopes) as an internal standard for 1 h at 37°C. Following lysis, a standard curve of dilution of D3-creatine was added to the last row of each plate. Plates were centrifuged for 5 min and supernatant was diluted 1:2 in H_2O. Sample desalting was performed on a high throughput Agilent RapidFire-365. From the analytical injection plate, approximately 40 µL of sample was desalted through an Agilent RapidFire Cartridge Graphitic Carbon Type D (G9206A) using water with 0.1% ammonium hydroxide as the loading mobile phase A for 3000 milliseconds at a flow rate of 1.5 mL/min. The sample was then eluted from the cartridge using 80% acetonitrile containing 0.1% ammonium hydroxide for a total time of 3000 milliseconds at a flow rate of 1.25 mL/min. An Agilent Q-ToF 6545 was used as the analyzing mass spectrometer via electrospray ionization. Capillary voltage was set to 3 kV, with a gas temperature of 350°C. Nitrogen drying gas was applied at 12 L/min with a pressure of 40 psi, and sheath gas was set to 375°C with a flow of 12 L/min. All data were collected with a positive ion polarity and stored as centroid data. Agilent MassHunter QToF was

used as the integration software for measuring area under the peak for both D3-creatine and D5-creatine as the internal standard. A linear regression was used from a standard curve of D3-creatine to calculate absolute concentration from peak area.

SLC7A5 isoleucine transport assay

Following a 24 h induction with tetracycline, cells were washed with sodium-free LCIS (20 mM HEPES, 140 mM choline chloride, 2.5 mM KCl, 1.8 mM $CaCl_2$, 1 mM $MgCl_2$, pH 7.4) before JNT-517 was added in 8-point concentration-response diluted in sodium-free LCIS. JPH203 (Selleckchem) was used as a positive control *(34)* at a concentration of 1.3 μM and DMSO was used as a negative control. Cells were incubated for 30 min at room temperature before a mixture of unlabeled L-isoleucine (Millipore Sigma) and 10 μCi/mL 4,5-3H(N)-labeled L-isoleucine (American Radiolabeled Chemicals) was added at a concentration of 50 μM. After a 2 min incubation at room temperature, cells were washed once with sodium-free LCIS and lysed in scintillation fluid (PerkinElmer). The uptake of radioactive substrate was measured on a Microbeta^2 microplate counter (PerkinElmer).

Fig S1 RAPID schematic. (A) A reactive affinity probe (RAP) consists of a binding element, a reactive moiety and a reporter group. (B) A cell line expressing the epitope-tagged target of interest is incubated with a RAP that binds to the target of interest via its binding element. The RAP is covalently bound to the target following UV crosslinking of its reactive moiety. Cells are lysed and the target of interest is captured on antibody-coated plates via the epitope tag. Biotin (B) is conjugated to the RAP's reporter group in a click reaction and the biotinylated RAP is

detected by ELISA tracer-tagged streptavidin in an electrochemiluminescent reaction. In a RAPID high-throughput screen, cells expressing the target of interest are co-incubated with a RAP and a library of small molecules. Small molecules with affinity to the target of interest may compete with the RAP for binding.

Fig S2 JNT-517 shows selectivity against SLC1A5, SLC6A8 and SLC7A5. Dose-response curves of JNT-517 in in Flp-In T-REx 293 cells overexpressing SLC1A5 (indigo), SLC6A8 (purple) or co-expressing SLC7A5 and SLC3A2 (yellow). Error bars denote S.D. from two technical replicates in a single experiment.

Fig S3 Urinary amino acid excretion in *Pah^{enu2}* mice treated with JN-170. 12-hour urine samples were collected in metabolic cages following a single dose administration of vehicle (n=24) or 50 – 250 mg/kg JN-170 (n=4-6 per group). Data represent mean ± S.D.

Fig S4 JN-170 pharmacokinetics and plasma amino acid levels in Pahenu2 and C57Bl/6 mice. (A) JN-170 pharmacokinetics in WT mice (C57Bl/6, indigo) or *Pah^{enu2}*-BTBR mice (purple) were orally administered 200 mg/kg JN-170. JN-170 plasma exposure was comparable between strains between 0 – 3 h (light blue box). (B) Plasma Gln and Phe in WT mice or *Pah^{enu2}*-BTBR mice treated with vehicle or 200 mg/kg JN-170 3 h post dose. N=5-6 for JN-170 treatment groups (WT/*Pah^{enu2}*), N=24 for vehicle-treated *Pah^{enu2}* mice.

Fig S5 Healthy volunteer study design and disposition. (A) SAD and MAD study design. There were 8 planned participants per cohort (6 active drug, 2 placebo). Part A, single ascending dose; Part B, multiple ascending doses.

Fig S6 Urinary amino acid excretion correlates with plasma amino acid concentration in healthy volunteers administered JNT-517. Total 8-hour urinary amino acid excretion following a single 170 mg dose of JNT-517. Data represent the average amino acid excretion across subjects (n=6), plotted over the average pre-dose plasma amino acid concentration. Error bars denote S.D.

Supplementary table 1 Baseline characteristics of SAD and MAD cohorts. Table A: baseline characteristics of SAD cohort; Table B: baseline characteristics of MAD cohort. BMI, body mass index; SD, standard deviation

Supplementary table 2 Summary of JNT-517 PK parameters in the SAD cohorts. AUC_{0-inf}/D, area under the curve from timepoint zero to infinity normalized to dose level; Cl/F, ratio of clearance to bioavailability; Cmax, maximum measured concentration normalized to dose level; Geomean, geometric mean; $T_{1/2}$, drug half life; T_{max}, time at which Cmax is reached

Supplementary table 3 Summary of MAD subjects with plasma amino acid concentrations below lower limit of normal. Total number of subjects and percent of total cohort with plasma amino acid concentrations below lower limit of normal before dosing and after dosing. After

dosing period includes all measured time points to final plasma draw. BID, twice a day: PBO, placebo; QD, once a day

Fig S1

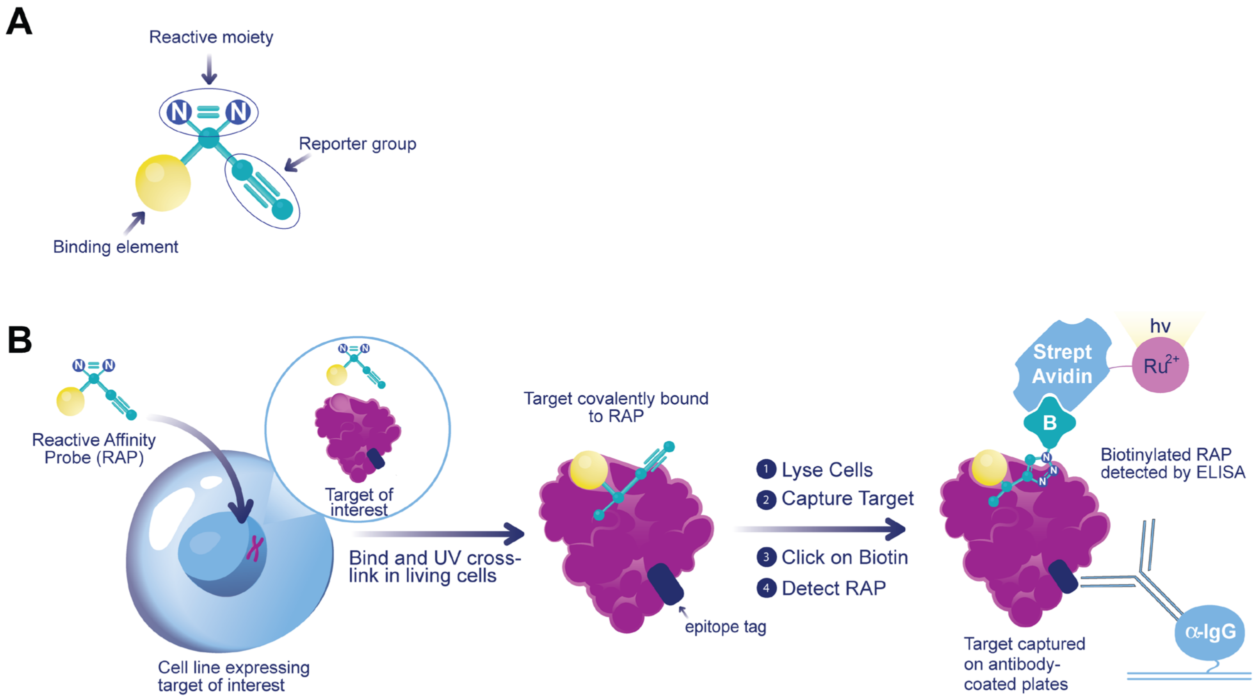

Fig S2

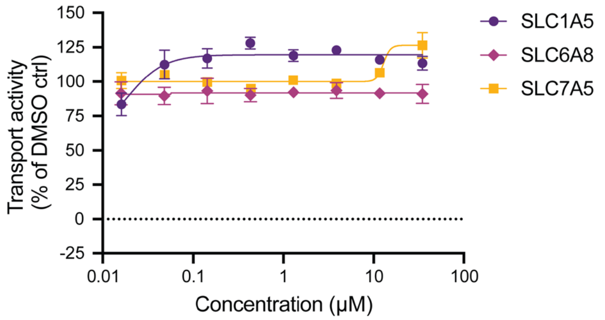

150
125
100
75
50
25
0
-25
Transport activity
(% of DMSO ctrl)
0.01
0.1
1
10
100
Concentration (μM)
SLC1A5
SLC6A8
SLC7A5

Fig S3

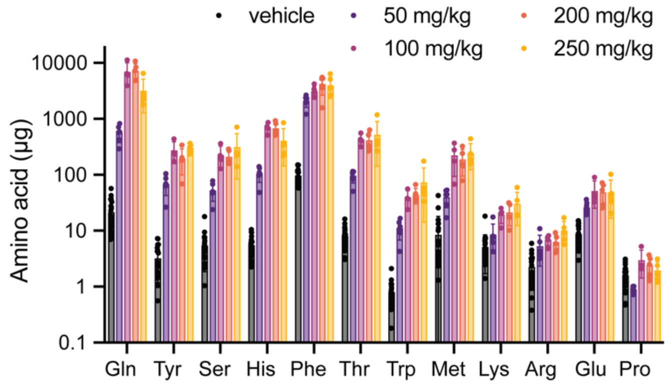

vehicle
50 mg/kg
100 mg/kg
200 mg/kg
250 mg/kg
10000
1000
100
10
1
0.1
Amino acid (μg)
Gln
Tyr
Ser
His
Phe
Thr
Trp
Met
Lys
Arg
Glu
Pro

Fig S4

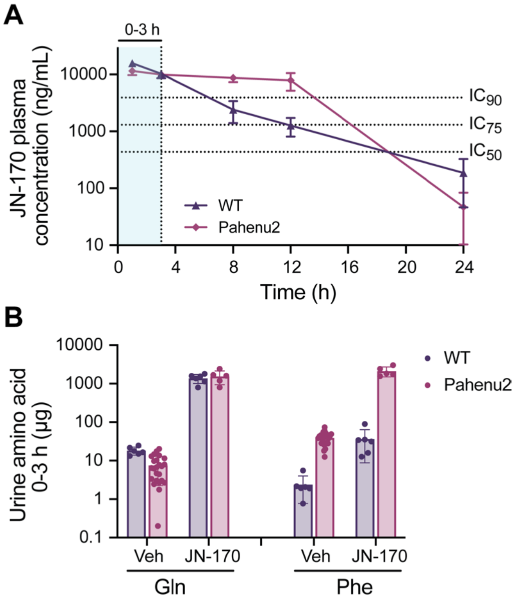
A
0-3 h
JN-170 plasma concentration (ng/mL)
10000
1000
100
10
IC90
IC75
IC50
WT
Pahenu2
0
4
8
12
16
20
24
Time (h)
B
Urine amino acid 0-3 h (µg)
10000
1000
100
10
1
0.1
WT
Pahenu2
Veh
JN-170
Veh
JN-170
Gln
Phe

Fig S5

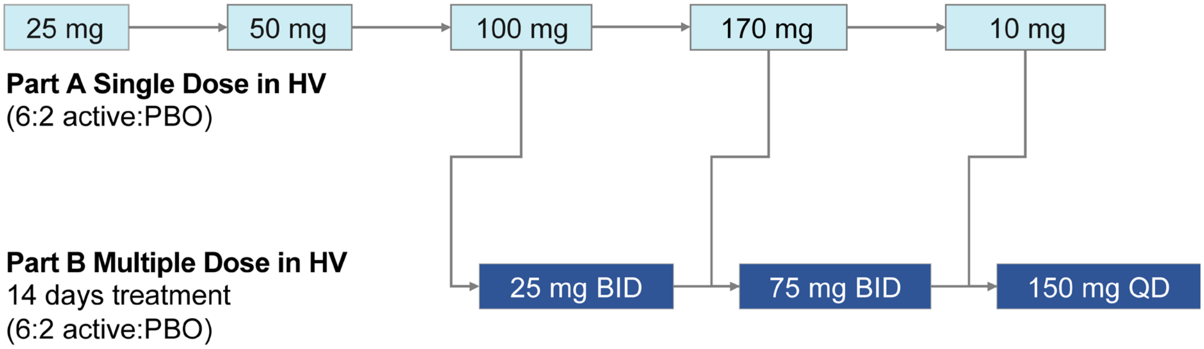
25 mg
50 mg
100 mg
170 mg
10 mg
Part A Single Dose in HV
(6:2 active:PBO)
Part B Multiple Dose in HV
14 days treatment
(6:2 active:PBO)
25 mg BID
75 mg BID
150 mg QD

Fig S6

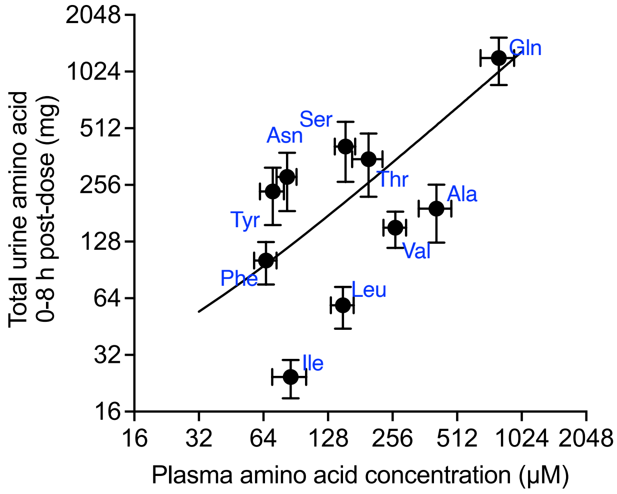
Total urine amino acid
0-8 h post-dose (mg)
2048
1024
512
256
128
64
32
16
Gln
Ser
Asn
Thr
Ala
Tyr
Val
Phe
Leu
Ile
16
32
64
128
256
512
1024
2048
Plasma amino acid concentration (μM)

Supplementary Table 1 Baseline characteristics of (A) SAD and (B) MAD cohorts

**Table A**	**JNT517 10 mg N = 6**	**JNT517 25 mg N = 6**	**JNT517 50 mg N = 6**	**JNT517 100 mg N = 6**	**JNT517 170 mg N = 6**	**Placebo N = 10**
**Age**<br>mean in years (SD)	32.8 (13.2)	29.5 (9.0)	24.0 (3.2)	26.3 (5.6)	33.3 (12.2)	28.2 (7.8)
**Gender**<br>Number Male, Female	3, 3	4, 2	4, 2	5, 1	5, 1	4, 6
**Race**<br>Number (%)<br>White<br>Asian<br>Black	5 (83.3%)<br>1 (16.7%)	5 (83.3%)<br>1 (16.7%)	4 (66.7%)<br>1 (16.7%)<br>1 (16.7%)	6 (100%)	5 (83.3%)<br>1 (16.7%)	7 (70%)<br>3 (30%)
**BMI**<br>mean (SD)	25.5 (2.9)	24.5 (4.6)	25.7 (4.6)	23.4 (2.4)	27.2 (4.1)	24.8 (4.2)

**Table B**	**JNT517<br>25 mg BID<br>N = 6**	**JNT517<br>75 mg BID<br>N = 6**	**JNT517<br>150 mg QD<br>N = 6**	**Placebo<br><br>N = 6**
**Age**<br>mean in years (SD)	31.2 (9.5)	22.3 (2.9)	26.8 (7.1)	26.2 (5.6)
**Gender**<br>Number Male, Female	4, 2	4, 2	3, 3	4, 2
**Race**<br>Number (%)<br>White<br>Asian<br>Multiple	6 (100%)	6 (100%)	4 (66.7%)<br>1 (16.7%)<br>1 (16.7%)	6 (100%)
**BMI**<br>mean (SD)	24.8 (2.6)	25.3 (5.2)	28.0 (6.2)	22.9 (1.7)

Supplementary Table 2 Summary of JNT-517 PK parameters in the SAD cohorts

	10 mg	25 mg	50 mg	100 mg	170 mg
**Geomean C_{max}/D (ng/mL/mg) [%CV]**	45.9 [23.6]	48.5 [25.5]	44.6 [14.8]	35.0 [13.0]	32.9 [24.9]
**Geomean $AUC_{0\text{-}inf}$/D (ng*h/mL/mg) [%CV]**	340 [66.0]	402 [59.0]	348 [34.5]	239 [42.4]	269 [33.6]
**Median Tmax (h) [min, max]**	0.75 [0.5, 1.07]	1.0 [0.5, 2.0]	0.75 [0.5, 2.0]	1.0 [0.5, 1.5]	0.75 [0.5, 2.0]
**Mean $T_{1/2}$ (h) [%CV]**	6.37 [50.2]	7.78 [50.7]	6.17 [35.4]	5.89 [31.9]	6.04 [24.7]
**Mean Cl/F (L/h) [SD]**	3.36 [1.75]	2.78 [1.27]	3.00 [0.873]	4.52 [2.11]	3.87 [1.15]

Supplementary Table 3 Summary of MAD subjects with plasma amino acid concentrations below lower limit of normal

Amino acid (mmol/L)	Ref. range (mmol/L)	PBO		25 mg BID		75 mg BID		150 mg QD	
		Pre-dose	Post-dose	Pre-dose	Post-dose	Pre-dose	Post-dose	Pre-dose	Post-dose
**Alanine**	120 - 580	0 (0.0%)	0 (0.0%)	0 (0.0%)	0 (0.0%)	0 (0.0%)	0 (0.0%)	0 (0.0%)	0 (0.0%)
**Arginine**	25-130	0 (0.0%)	0 (0.0%)	0 (0.0%)	0 (0.0%)	0 (0.0%)	0 (0.0%)	0 (0.0%)	0 (0.0%)
**Asparagine**	30 – 85	0 (0.0%)	0 (0.0%)	0 (0.0%)	0 (0.0%)	0 (0.0%)	0 (0.0%)	0 (0.0%)	0 (0.0%)
**Cystine**	5 - 60	**4 (66.7%)**	**4 (66.7%)**	**5 (83.3%)**	**5 (83.3%)**	0 (0.0%)	0 (0.0%)	**6 (100.0%)**	**5 (83.3%)**
**Glutamate**	20 – 160	0 (0.0%)	0 (0.0%)	0 (0.0%)	0 (0.0%)	0 (0.0%)	0 (0.0%)	0 (0.0%)	0 (0.0%)
**Glutamine**	300 - 750	0 (0.0%)	0 (0.0%)	0 (0.0%)	0 (0.0%)	0 (0.0%)	0 (0.0%)	0 (0.0%)	0 (0.0%)
**Glycine**	110 - 450	0 (0.0%)	0 (0.0%)	0 (0.0%)	0 (0.0%)	0 (0.0%)	0 (0.0%)	0 (0.0%)	0 (0.0%)
**Isoleucine**	40 - 135	0 (0.0%)	0 (0.0%)	0 (0.0%)	0 (0.0%)	0 (0.0%)	0 (0.0%)	0 (0.0%)	0 (0.0%)
**Leucine**	60 – 200	0 (0.0%)	0 (0.0%)	0 (0.0%)	0 (0.0%)	0 (0.0%)	0 (0.0%)	0 (0.0%)	0 (0.0%)
**Lysine**	70 – 290	0 (0.0%)	0 (0.0%)	0 (0.0%)	0 (0.0%)	0 (0.0%)	0 (0.0%)	0 (0.0%)	0 (0.0%)
**Methionine**	10 - 50	0 (0.0%)	0 (0.0%)	0 (0.0%)	0 (0.0%)	0 (0.0%)	0 (0.0%)	0 (0.0%)	0 (0.0%)
**Phenylalanine**	30 – 100	0 (0.0%)	0 (0.0%)	0 (0.0%)	0 (0.0%)	0 (0.0%)	0 (0.0%)	0 (0.0%)	0 (0.0%)
**Proline**	90 - 400	0 (0.0%)	0 (0.0%)	0 (0.0%)	0 (0.0%)	0 (0.0%)	0 (0.0%)	0 (0.0%)	0 (0.0%)
**Serine**	70 - 300	0 (0.0%)	0 (0.0%)	0 (0.0%)	0 (0.0%)	0 (0.0%)	0 (0.0%)	0 (0.0%)	**1 (16.7%)**
**Threonine**	60 - 280	0 (0.0%)	0 (0.0%)	0 (0.0%)	0 (0.0%)	0 (0.0%)	0 (0.0%)	0 (0.0%)	0 (0.0%)
**Tyrosine**	40 - 130	0 (0.0%)	0 (0.0%)	0 (0.0%)	0 (0.0%)	**1 (16.7%)**	0 (0.0%)	0 (0.0%)	**2 (33.3%)**
**Valine**	130 - 360	0 (0.0%)	0 (0.0%)	0 (0.0%)	0 (0.0%)	0 (0.0%)	0 (0.0%)	0 (0.0%)	0 (0.0%)

Supplementary References

28. Hasan M, Ferguson A. Measurements of intestinal villi non-specific and ulcer-associated duodenitis-correlation between area of microdissected villus and villus epithelial cell count. *J Clin Pathol*. 1981;34(10):1181–1186.

29. Mayersohn, Michael. *Modern Pharmaceutics Volume 1: Basic Principles and Systems, Fifth Edition, Chapter 2: Principles of Drug Absorption*. CRC Press
